# 

**Published:** 1893-08

**Authors:** 


					﻿Vol XIII.
AUGUST, 1893.
No. 8.
THE
HOMEOPATHIC pHY^lClAJtf,
A Monthly Journal of Homoeopathic Materia Medica
and Clinical Medicine.
“ He is a freeman whom truth makes free, and all are slaves beside."
“ Seek the Truth : come whence it may, cost what it will."
EDITED AND PUBLISHED BY
WALTER M. JAMES, M. D.
PHILADELPHIA :
No. 1125 SPRUCE 8TREET.
x. eras'» o ar:
ALFRED HEATH & CO., Bole AOKKTS FOB Obkat Bbitaim,
114 Ebury Street, Eaton Square.
43**Feorty Subscription, $2.SO, invariably in advance. Single numbers, 2S etc.
Entered at the Philadelphia Peal-Offiee aa SeMeid Claaa Mall Matter.
				

